# Supplementary material for: Estimating conformational landscapes from Cryo-EM particles by 3D Zernike polynomials
Source: Nat Commun. 2023 Jan 11;14:154. doi: 10.1038/s41467-023-35791-y (PMC9832421; doi:10.1038/s41467-023-35791-y)
Supplement: Supplementary file 1 — Supplementary Information [file 41467_2023_35791_MOESM1_ESM.pdf]

Supplementary Information

Estimating conformational landscapes from Cryo-EM particles by 3D Zernike polynomials

D. Herreros*[1], R.R. Lederman[2], J.M. Krieger[1], A. Jiménez-Moreno[1], M. Martínez[1], D. Myška[3], D. Strelak[1,4], J. Filipovic[3], C.O.S. Sorzano[1], and J.M.Carazo[1]

[1]Centro Nacional de Biotecnologia-CSIC, C/ Darwin, 3, 28049, Cantoblanco, Madrid, Spain
[2]The Department of Statistics and Data Science, Yale University, New Haven, CT, USA
[3]Institute of Computer Science, Masaryk University, Botanická 68a, 60200 Brno, Czech Republic
[4]Faculty of Informatics, Masaryk University, Botanická 68a, 60200 Brno, Czech Republic

* Corresponding author
E-mail: dherreros@cnb.csic.es

Supplementary Methods

A 3D Real-valued generalized Zernike polynomials

The Zernike3D basis is an infinite funcitonal space able to reproduce any function within the unit ball B. Therefore, it is convinient to define the basis components as a composition of a radial and an angular function.

In general, the expansion of any real valued function $g(\boldsymbol{r}) \in L_2(B)$ in this basis is defined by the formula

$$g(\boldsymbol{r}) = \sum_{l=0}^{\infty}\sum_{n=0}^{\infty}\sum_{m=-l}^{l} \boldsymbol{\alpha}_{l,n,m} Z_{l,n,m}(\boldsymbol{r}). \tag{1}$$

where $\boldsymbol{\alpha}_{l,n,m}$ are real-valued coefficients, and the $Z_{l,n,m}(\boldsymbol{r})$ are the 3D real-valued (normalized) generalized Zernike polynomials defined by the formula

$$Z_{l,n,m}(\boldsymbol{r}) = \bar{R}^{1}_{l,n}(r) y_l^m(\theta, \phi) \tag{2}$$

where r is the radial component of the 3D coordinate $\boldsymbol{r}$, and θ and ϕ are its polar and azimuthal angles in spherical coordinates, n and l are non-negative integers, and m is an integer such that $-l \leq m \leq l$.

As it can be seen from 2, we decompose the Zernike3D basis into a radial and an angular component. A logical choice of functions to define the angular component are the real-valued spherical harmonics y_l^m, that depend on the *spherical frequency* l as follows:

$$y_l^m(\theta,\phi) = (-1)^m \sqrt{\frac{2l+1}{4\pi}\frac{(l-|m|)!}{(l+|m|)!}} P_l^{|m|}(cos\theta) \begin{cases} 1 & \text{if } m = 0 \\ \sqrt{2}cos(m\phi) & \text{if } m > 0 \\ \sqrt{2}sin(|m|\phi) & \text{if } m < 0 \end{cases} \tag{3}$$

where P_l^m are the associated Legendre polynomials defined by the formula

$$P_l^m(x) = \frac{(-1)^m}{2^l l!}(1-x^2)^{m/2}\frac{d^{l+m}}{dx^{l+m}}(x^2-1)^l. \tag{4}$$

Our radial component consist of the (normalized) generalized Zernike polynomials (applied to the 3D case) defined as:

$$\bar{R}^p_{l,n}(x) = \sqrt{2}\sqrt{2n+l+\frac{p}{2}+1} R^p_{l,n}(x). \tag{5}$$

being $R^p_{l,n}(x)$ the unnormalized generelized Zernike polynomials

$$R^p_{l,n}(x) = (-1)^n x^l P_n^{(l+\frac{p}{2},0)}\left(1-2x^2\right), \tag{6}$$

The main reason to choose the normalized version of the function is to make them orthonormal with respect to the inner product determined by p. The previous parameter is related with the dimensionality and inner product of the ball where the basis is defined. Therefore, fix the value $p = 1$ to yield an orthonormal basis with respect to the natural inner product on $L_2(B)$,

which corresponds to the case of a 3D ball.

We would like to remark that our choice of functions to define the basis is not unique. However, the combination of the normalized generalized Zernike polynomials and the spherical harmonics yields a basis with very helpful properties to manipulate the computed deformation fields, such us being closed under rotations.

B Closure under rotations

As is well-known, the rotation of the frame of reference of spherical harmonics of a given spatial frequency l is a unitary operation and closed rotations. If follows that the linear combination of $\sum_{n=0}^{N}\sum_{l=0}^{L}\sum_{m=-l}^{l}\boldsymbol{\alpha}_{l,n,m}Z_{l,n,m}(\boldsymbol{r})$ is closed under rotations. In other words, regardless of the frame of axis we choose for our spherical harmonics, we can represent the same functions using our choice of basis.

The deformation field $\boldsymbol{g}_L(\boldsymbol{r})$ expressed by the Zernike3D basis is a 3D vector defined at every position $\boldsymbol{r}$. Thus, any rotation applied to axes x, y, z will be propagated to the deformation vectors $\boldsymbol{g}_L(\boldsymbol{r})$, defined now in the new coordinate system. As is well known,

$$A\boldsymbol{g}_L(\boldsymbol{A^{-1}r}) = \sum_{n=0}^{N}\sum_{l=0}^{L}\sum_{m=-l}^{l} A \begin{pmatrix} \alpha^x_{l,n,m} \\ \alpha^y_{l,n,m} \\ \alpha^z_{l,n,m} \end{pmatrix} \tilde{Z}_{l,n,m}(\boldsymbol{A^{-1}r}) \tag{7}$$

where A is the appropriate unitary rotation matrix.

From the previous reasoning, it follows that the Zernike3D basis is closed under rotations; As long as the origin of the reference frame is kept fixed, we can find a new set of coefficients $\boldsymbol{\alpha}'_{l,n,m}$ that represents the same deformation independently of the reference frame of rotation. Furthermore the transformation between frames of reference is unitary.

It is worth mentioning that the previous property is only valid for continuous spaces. Since we are working with discretized volumes, the basis does not fulfill completly the closue under rotations property. However, the property can still be applied as long as the application carefully considers the discretization of the space.

C Conformational landscape of simulated chaperone CCT data

In order to show the capabilities and describe the characteristics of the continuous heterogeneity analysis carried out by the Zernike3D algorithm, a synthetic dataset based on five simulated conformations of a CCT complex was studied.

The dataset was computationally created by the NMA-based (Normal Mode Analysis) approach referred to as adaptive ANM (Anisotropic Network Model) (1) implemented in ProDy software (2). The dataset was designed to reproduce the open-close transition described in (3).

We obtained a sampling of 5 structures along the previously used conformational transition (7). Subsequently, each atomic structure was converted to Coulomb potential maps using the Electron Atomic Scattering Factors (EASFs) (5), and a projection gallery of 45 particles was created for each conformation, leading to a total of 225 particles (having such a low number of particles was done on purpose to show that we can successfully analyze small datasets where a genuine continuous flexibility is present). The previous phantom particles were not further process to include noise, shifts, or the Contrast Transfer Function (CTF) to be able to determine the lower bound of the method's errors. In our tests with experimental data the effects of noise, shifts and CTF are obviously included.

For this test, a set of coefficients $\alpha_{l,n,m}$ was obtained for each one of the 225 particles. The basis degrees chosen were $N = 3$ and $L = 2$, leading to coefficient vectors of 39 different components each. Thus, the dimensions of the coefficients space must be reduced before its visualization with a dimensionality reduction method such as UMAP (Uniform Manifold Approximation and Projection) (4) or PCA (Principal Components Analysis) (6). The reduced coefficient space from PCA is shown in Figure 1a.

As seen from the resulting embedding, the first three conformational changes are well differentiated. However, the images corresponding to the last two changes tend to be placed in a similar region of the space. As we showed in our previous work (7), the changes corresponding to the last structures are mediated by high-frequency modes, making them more similar and less resolvable in the coefficient space.

As we explained in Methods Subsection *"Merging embeddings of different nature"* in the main manuscript, it is possible to translate both particles and maps into Zernike3D coefficients. Therefore, it is possible to determine where the maps are located in the space defined by the particles to identify each conformation better. Figure 1b shows the combined coefficient space between the maps simulated from the atomic structures and the images projected from these maps.

Figure 1b provides valuable information about the landscapes computed with the Zernike3D approach.

- Since the method relies on a reference map, the estimation error for each particle will increase as the conformation between the particle and the reference differs. The main reason behind this effect is that we are trying to estimate a per-particle conformation. Therefore, the Zernike3D approach can use the information along the image plane to define the deformation field needed to reach a new state. However, it cannot estimate this information along the projection direction, as it has been collapsed due to the projection process. As a result, we can see a higher dispersion in Figure 1b as particle move away from the reference map.

- The Zernike3D coefficient estimated for a particle will be placed surrounding the real 3D state associated with that particle. If we focus on the white dots in Figure 1b (corresponding to the real 3D maps used to generate the projected particles), we can appreciate the previous arrangement of the per-particle Zernike3D coefficients. As expected, conformations closer to the reference will be closer to the real conformation, as the error committed along the projection direction will be smaller.

It is worth to highlight that the previous effects are only significant when the signal to noise ratio of the particles is high. Thus, in a real case scenario, the large amount of noise present in every particle will dominate.

Since the Zernike3D approach estimates conformational changes based on the information of a single particle, it was interesting to test the capability of the method to determine meaningful landscapes at different levels of noise. To that end, we simulated a larger dataset of 700 particles with the simulated CCT maps described before. Gaussian noise was posteriorly added to the particle images to simulate new datasets with varying Signal to Noise Ratios (SNRs). Each one of the previous datasets were subjected to the Zernike3D analysis to assess the capacity of the method to recover conformational landscapes under different noise levels. The resulting landscapes are provided in Figure 4.

As expected, higher noise levels will progressively decrease the quality of the landscapes. However, it is possible to see that for common SNRs in CryoEM (0.01-0.001), the Zernike3D approach is still able to recover appropriately a landscape with the expected shape and order of the conformations.

Lastly, we applied the estimated coefficients to reconstruct a map with the new ART algorithm described previously. The main objective of this test is to determine whether deformation coefficients can be used to recover the reference volume from those images projected from the other conformations present in the dataset. Due to the reduced number of particles, the reconstructed map is not expected to achieve high resolution. Instead, the objective of this phantom dataset was to prove that ZART can "undo" per-particle conformational changes and achieve better resolution independently of the states they represent.

The comparison of the resulting reconstruction and the reference volume is shown in Figure 2. The figure shows that the new ZART reconstruction method can revert properly image deformations, recovering the different reference volume from them.

Supplementary References

[1] Z. Yang, P. Májek, and I. Bahar. Allosteric transitions of supramolecular systems explored by network models: Application to chaperonin GroEL. *PLOS Compututational Biology*, 5(4):1–21, 2010.

[2] S. Zhang, J.M. Krieger, Y. Zhang, C. Kaya, B. Kaynak, K. Mikulska-Ruminska, P. Doruker, H. Li, and I. Bahar. ProDy 2.0: Increased scale and scope after 10 years of protein dynamics modelling with python. *Bioinformatics*, 37(20):3657–3659, 2021.

[3] Y. Cong, G.F. Schröder, A.S. Meyer, J. Jakana, B. Ma, M.T. Dougherty, M.F. Schmid, S. Reissmann, M. Levitt, S.L. Ludtke, J. Frydman, and W. Chiu. Symmetry-free cryo-EM structures of the chaperonin TRiC along its ATPase-driven conformational cycle. *EMBO Journal*, 31(3):720–730, 2012.

[4] L. McInnes, J. Healy, N Saul, and L. Großberger. Umap: Uniform manifold approximation and projection. *Journal of Open Source Software*, 3(29):861, 2018.

[5] C.O.S. Sorzano, J. Vargasm J. Oton, V. Abrishami, J.M. de ka Rosa-Trevin, A. Fernandez-Alderete, C. Martinez-Rey, R. Marabini, and J.M. Carazo Fast and accurate conversion of atomic models into electron density maps *AIMS Biophysics*, 2(1):8–20, 2015.

[6] I. Jolliffe and J. Cadima. Principal component analysis: A review and recent developments. *Philosophical Transactions of the Royal Society A: Mathematical, Physical and Engineering Sciences*, 374:20150202, 2016.

[7] D. Herreros, R.R. Lederman, J. Krieger, A. Jiménez-Moreno, M. Martínez, D. Myška, D. Strelak, J. Filipovic, I. Bahar, J.M. Carazo, and C.O.S. Sorzano. Approximating deformation fields for the analysis of continuous heterogeneity of biological macromolecules by 3D Zernike polynomials. *IUCrJ*, 8(6):992–1005, 2021.

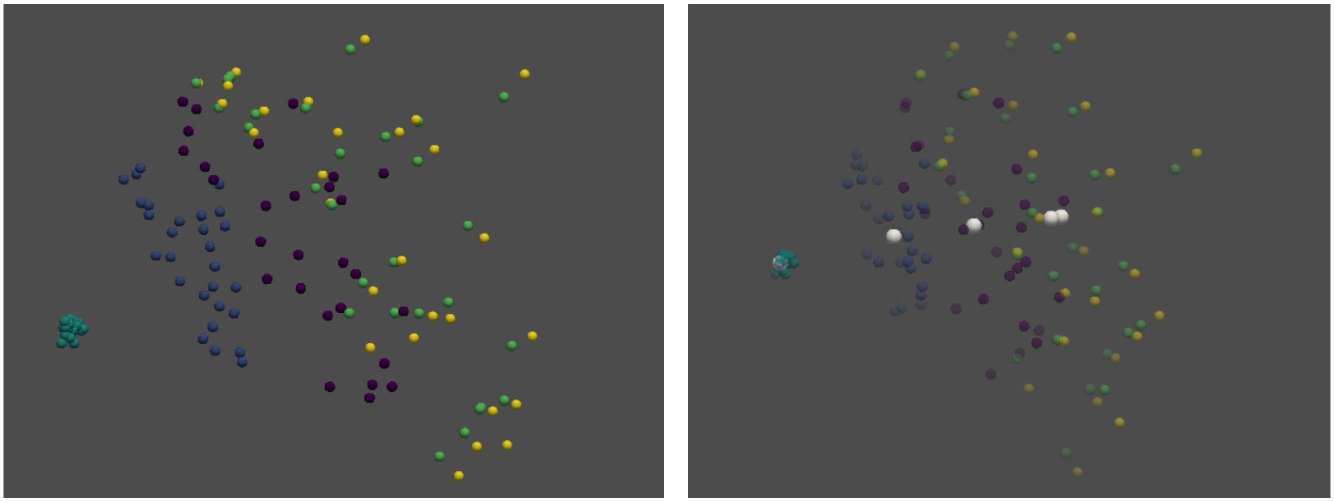

(a) (b)

Suplemmentary Fig. 1: a) PCA representation of the Zernike3D coefficient space for particles representing the open-closed transition of a chaperone CCT complex simulated by adaptive ANM. Each point in the Figure represents a different projection of a volume, and the coloring determines to which conformation each image belongs. The coefficient space shows a clear distintion among the different sampled conformations of the CCT complex. b) PCA representation of the Zernike3D coefficient space resulting from the translation of both projections and maps to Zernike3D coefficients. The white dots represent the coefficients arising from the deformation fields of the maps that gave rise to the different projections shown in colors. The results show that the maps tend to be surrounded by their respective projection images.

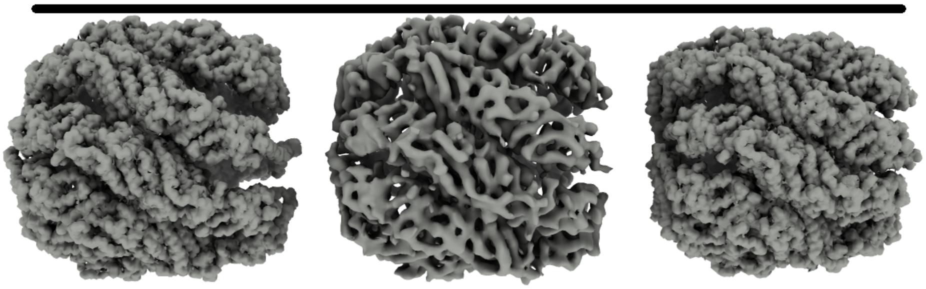

Suplemmentary Fig. 2: New ZART reconstruction method applied to the embedding shown in Figure 1. The map to the left corresponds to the reference open map, the map in the middle shows the ZART reconstruction recovered from the projections not associated to the reference volume (a total of 180 particles corresponding to the blue, purple, green, and yellow dots displayed in the coefficient space), and the map to the right is one of the original closed conformations whose projections were used to reconstruct the ZART map. The comparison of the volumes shows that the reference map is appropriately recovered when the conformation of each projection is undeformed by the deformation fields estimated with the Zernike3D algorithm. The middle map is unsurprisingly of low resolution in line with the small number of particles. The black line is provided to highlith the open-closed conformational change represented by the maps.

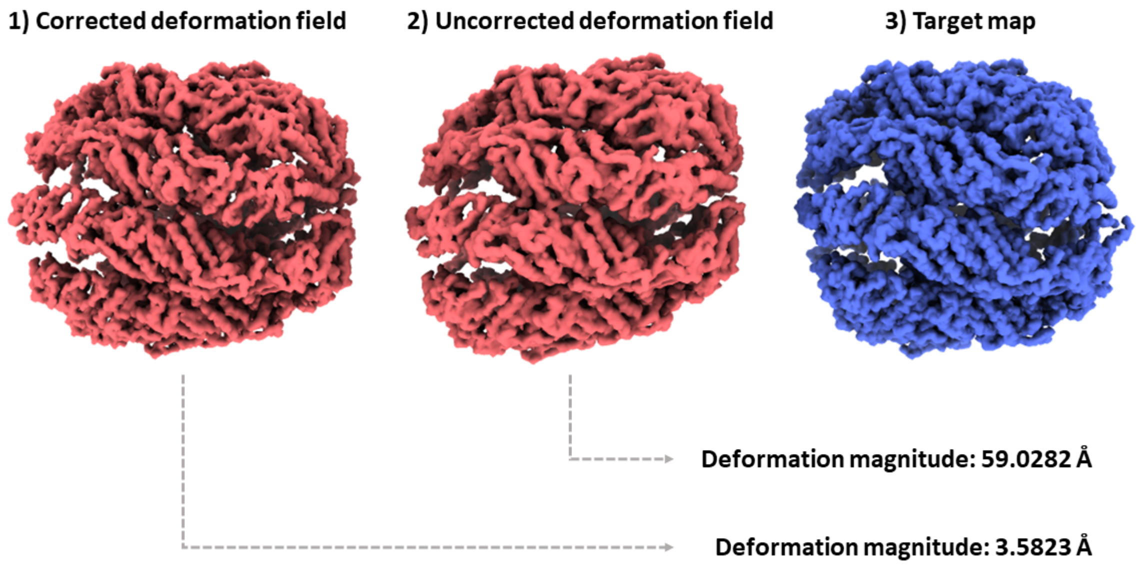

Suplemmentary Fig. 3: Example of deformation field inconsistency along the projection direction. Pannel 1) shows the deformed reference map after correcting the aberrations associated with the deformation field along the projection direction. Pannel 2) shows the deformed reference map after being modified by the original deformation field (no correction along the projection direction). Pannel 3) shows the target map the deformation field is trying to approximate. As it can be seen from the Figure, if the projection direction is not handled correctly, the estimation over missing information will lead to unwanted conformational changes with exagerated deformation magnitudes.

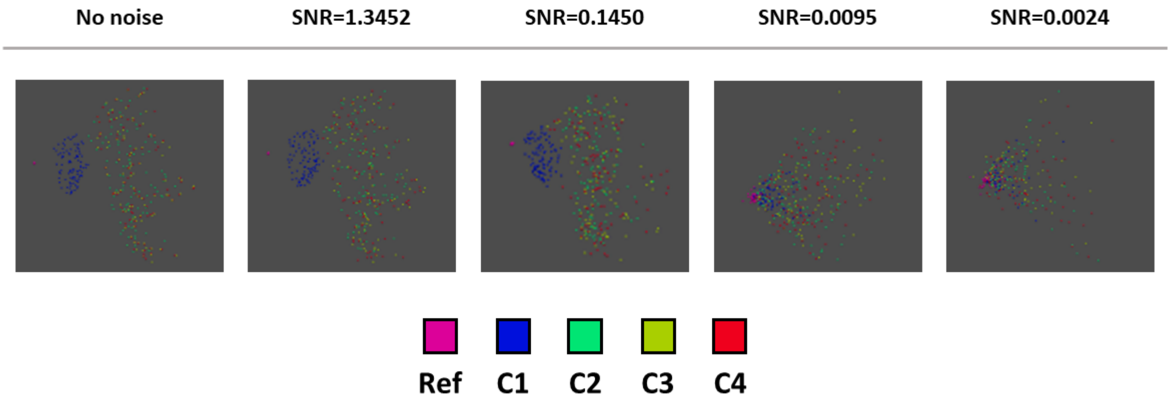

Suplemmentary Fig. 4: Assessment of the robustness of the Zernike3D algorithm to different levels of noise. Even at low SNRs, the Zernike3D landscape shows a meaningful shape and order of the conformations described by the phantom particles.
