# Supplementary material for: Estimating conformational landscapes from Cryo-EM particles by 3D Zernike polynomials
Source: Nat Commun. 2023 Jan 11;14:154. doi: 10.1038/s41467-023-35791-y (PMC9832421; doi:10.1038/s41467-023-35791-y)
Supplement: Supplementary file 3 — Description of Additional Supplementary Files [file 41467_2023_35791_MOESM3_ESM.pdf]

Description of Additional Supplementary Files

File Name: Supplementary Movie 1
Description: Morphing of 10 conformational states of the *P. falciparum* 80S ribosome recovered from the Zernike3D conformational landscape presented in Figure 1. Each conformation has been generated at map and atomic level thanks to the application of the Zernike3D deformation fields.

File Name: Supplementary Movie 2
Description: Morphing of the 5 spliceosomal conformational states presented in Figure 4b and Figure 4c. The states were extracted from the Zernike3D conformational landscape estimated from the EMPIAR-10180 particles, also provided in Figure 4a. Thanks to the application of the Zernike3D deformation fields, each state was generated at both, map and atomic level.

File Name: Supplementary Movie 3
Description: Morphing of 20 different states of the SARS-CoV-2 spike in 1Up conformation obtained from the Zernike3D landscape provided in Figure 6. Each state was generated through the application of the Zernike3D deformation fields to get a map and atomic representation of the different estimated conformations.

File Name: Supplementary Movie 4
Description: Morphing of 7 SARS-CoV-2 conformations recovered from the main transition present in the Zernike3D landscapes provided in Figure 6, The different conformations were manually selected, and used to recover the Zernike3D deformation fields applied to the spike reference map and atomic structure.
