# Supplementary material for: Estimating conformational landscapes from Cryo-EM particles by 3D Zernike polynomials
Source: Nat Commun. 2023 Jan 11;14:154. doi: 10.1038/s41467-023-35791-y (PMC9832421; doi:10.1038/s41467-023-35791-y)
Supplement: Supplementary file 8 — Reporting Summary [file 41467_2023_35791_MOESM8_ESM.pdf]

nature portfolio

Corresponding author(s): David Herreros Calero

Last updated by author(s): Dec 15, 2022

Reporting Summary

Nature Portfolio wishes to improve the reproducibility of the work that we publish. This form provides structure for consistency and transparency in reporting. For further information on Nature Portfolio policies, see our Editorial Policies and the Editorial Policy Checklist.

Statistics

For all statistical analyses, confirm that the following items are present in the figure legend, table legend, main text, or Methods section.

n/a	Confirmed	
☒	☐	The exact sample size (*n*) for each experimental group/condition, given as a discrete number and unit of measurement
☒	☐	A statement on whether measurements were taken from distinct samples or whether the same sample was measured repeatedly
☒	☐	The statistical test(s) used AND whether they are one- or two-sided<br>*Only common tests should be described solely by name; describe more complex techniques in the Methods section.*
☒	☐	A description of all covariates tested
☒	☐	A description of any assumptions or corrections, such as tests of normality and adjustment for multiple comparisons
☒	☐	A full description of the statistical parameters including central tendency (e.g. means) or other basic estimates (e.g. regression coefficient) AND variation (e.g. standard deviation) or associated estimates of uncertainty (e.g. confidence intervals)
☒	☐	For null hypothesis testing, the test statistic (e.g. *F*, *t*, *r*) with confidence intervals, effect sizes, degrees of freedom and *P* value noted<br>*Give P values as exact values whenever suitable.*
☒	☐	For Bayesian analysis, information on the choice of priors and Markov chain Monte Carlo settings
☒	☐	For hierarchical and complex designs, identification of the appropriate level for tests and full reporting of outcomes
☐	☒	Estimates of effect sizes (e.g. Cohen's *d*, Pearson's *r*), indicating how they were calculated

Our web collection on statistics for biologists contains articles on many of the points above.

Software and code

Policy information about availability of computer code

Data collection

All the data analyzed in the submitted manuscript can be downloaded from EMPIAR website freely. The entries associated with the data are: 10028, 10514, 10516, and 10180.

The phantom dataset analyzed in the supplementary material was created using the tools provided in Scipion (v3.0.13), which is also open-source and publicly available. The dataset has been uploaded to GitHub so it can be easily accesed and downloaded in case any user wants to reproduce the results discussed in the supplementary material.

Data analysis

All the datasets were analyzed inside Scipion (v3.0.13) through the following softwares: Xmipp (v3.22.11 - Iris), CryoSparc (v3.3.2), and Flexutils (v1.0.0). These three softwares were installed as plugins inside Scipion, allowing us to use the latest versions supported by those plugins.

Although the three softwares mentioned previously were the main ones executed to analyze the datasets presented in the manuscript, the following programs were also applied at some point in the the workflows: Prody (v2.2.0), ChimeraX (v1.3), BSoft (v2.1.3).

The algorithms presented along the manuscript require Xmipp and Flexutils to be installed in Scipion to be executed appropiately.

For manuscripts utilizing custom algorithms or software that are central to the research but not yet described in published literature, software must be made available to editors and reviewers. We strongly encourage code deposition in a community repository (e.g. GitHub). See the Nature Portfolio guidelines for submitting code & software for further information.

Data

Policy information about availability of data

All manuscripts must include a data availability statement. This statement should provide the following information, where applicable:

- Accession codes, unique identifiers, or web links for publicly available datasets
- A description of any restrictions on data availability
- For clinical datasets or third party data, please ensure that the statement adheres to our policy

The datasets analyzed with the Zernike3D algorithm and ZART are publicly available in EMPIAR under the entries: 10028, 10514, 10516, and 10180. The phantom dataset processed in the Supplementary Material is available in GitHub in the repository Zernike3D_Phantom_Data.

Human research participants

Policy information about studies involving human research participants and Sex and Gender in Research.

Reporting on sex and gender	This information has not been collected.
Population characteristics	This information has not been collected.
Recruitment	This information has not been collected.
Ethics oversight	This information has not been collected.

Note that full information on the approval of the study protocol must also be provided in the manuscript.

Field-specific reporting

Please select the one below that is the best fit for your research. If you are not sure, read the appropriate sections before making your selection.

☒ Life sciences ☐ Behavioural & social sciences ☐ Ecological, evolutionary & environmental sciences

For a reference copy of the document with all sections, see nature.com/documents/nr-reporting-summary-flat.pdf

Life sciences study design

All studies must disclose on these points even when the disclosure is negative.

Sample size	Not applicable
Data exclusions	No data was excluded from the analysis.
Replication	Not applicable
Randomization	Not applicable
Blinding	Not applicable

Reporting for specific materials, systems and methods

We require information from authors about some types of materials, experimental systems and methods used in many studies. Here, indicate whether each material, system or method listed is relevant to your study. If you are not sure if a list item applies to your research, read the appropriate section before selecting a response.

Materials & experimental systems

n/a	Involved in the study
☒	☐ Antibodies
☒	☐ Eukaryotic cell lines
☒	☐ Palaeontology and archaeology
☒	☐ Animals and other organisms
☒	☐ Clinical data
☒	☐ Dual use research of concern

Methods

n/a	Involved in the study
☒	☐ ChIP-seq
☒	☐ Flow cytometry
☒	☐ MRI-based neuroimaging

March 2021
